# Direct oral anticoagulants in children with giant coronary artery aneurysms from Kawasaki disease: a systematic review and meta-analysis

**DOI:** 10.3389/fcvm.2026.1777856

**Published:** 2026-04-10

**Authors:** Sze Kiat Alan Ong, Deepan Raj, Anika W Xuen Lee, Liang Shen, Kian Keong Poh, Pei Lin Koh, Swee Chye Quek

**Affiliations:** 1 Department of Paediatrics, Khoo Teck Puat—National University Children’s Medical Institute, National University Health System, Singapore, Singapore; 2 Department of Paediatrics, Yong Loo Lin School of Medicine, National University of Singapore, Singapore, Singapore; 3 Biostatistics Unit, Yong Loo Lin School of Medicine, National University of Singapore, Singapore, Singapore; 4 Department of Cardiology, National University Heart Center, Singapore, Singapore

**Keywords:** anticoagulation, bleeding, heart, Kawasaki disease, pediatric, thromboembolism

## Abstract

**Background:**

To evaluate the comparative efficacy and safety profile of direct oral anticoagulants (DOACs) vs. conventional anticoagulation in children with Kawasaki disease (KD)-associated Giant coronary artery aneurysms (GCAAs).

**Methods:**

Databases searched included PubMed (MEDLINE), Embase, Cochrane Central Register of Controlled Trials and ClinicalTrials.gov from conception until October 2025. Studies that (1) enrolled patients younger than 19 years with documented KD-associated GCAAs; (2) administered DOACs; (3) reported at least one efficacy/safety outcome, were included. Efficacy outcomes included thromboembolic events (coronary thrombosis, myocardial infarction, systemic thromboembolism). Safety outcomes included major or Clinically Relevant Non-Major (CRNM) bleeding. A random-effects model was used to estimate the pooled effects.

**Results:**

Five studies [two randomized clinical trials (RCTs), two observational studies and one prospective interventional trial] were included with a total of 594 patients [DOACs: 474 (79.8%); standard of care, SOC: 120 (20.2%)]. The overall risk ratio of thromboembolic events for patients on DOACs vs. SOC was not statistically significant [RR 0.27 (95% CI: 0.02–3.17); *p* = 0.29]. The estimated pooled major or CRNM bleeding event rate was similar [DOACs: Proportion 0.001 (95% CI: 0.00–0.01)] vs. SOC: Proportion 0.03 (95% CI: 0.01–0.07). In the two RCTs, the estimated risk ratio between DOACs vs. SOC was not statistically significant [RR 0.26 (95% CI: 0.05–1.49); *p* = 0.13].

**Conclusion:**

DOACs have potentially non-inferior efficacy and safety profiles compared to conventional agents, supporting the use of DOACs as a first-line anticoagulation strategy in one of childhood's most serious cardiovascular conditions.

**Systematic Review Registration:**

https://www.crd.york.ac.uk/PROSPERO/view/CRD420251004094, PROSPERO CRD420251004094.

## 1 Introduction

Kawasaki disease (KD) represents the leading cause of acquired heart disease in children across developed nations, with coronary artery abnormalities occurring in approximately 25% of untreated cases (1). Giant coronary artery aneurysms (GCAAs), defined as coronary artery diameters exceeding 8 mm or *Z*-scores of ≥10, constitute the most severe cardiovascular sequelae, affecting 0.25%–1% of KD patients despite recommended therapy (2). The rates of coronary artery stenosis or complete occlusion reached approximately 42% over a mean follow-up of 12.5 years, and up to 23% of patients with GCAAs experienced acute myocardial infarction. These vascular abnormalities confer substantial morbidity necessitating lifelong anticoagulation strategies (3, 4).

The unique pathophysiology of KD-associated coronary arteriopathy presents distinct considerations for anticoagulation therapy. Unlike atherosclerotic coronary disease, KD induces necrotizing arteritis with destruction of the internal elastic lamina, smooth muscle cell proliferation, and progressive luminal myofibroblastic proliferation (5). This vascular remodeling creates a prothrombotic milieu characterized by endothelial dysfunction, platelet activation, and altered hemodynamics within aneurysmal segments (6).

Furthermore, the developmental pharmacologic considerations in pediatric patients introduce additional complexity. These include age-related variations in drug absorption, distribution, metabolism, and elimination which influence anticoagulant dosing and monitoring requirements (7). The evolving hemostatic system throughout childhood, with physiologically lower levels of vitamin K-dependent factors and natural anticoagulants compared with adults, necessitates careful consideration of bleeding risk stratification and dose optimization (8, 9). However, the application of direct oral anticoagulants (DOACs) in KD-associated GCAAs remains inadequately investigated, with limited comparative effectiveness data against conventional anticoagulation (9).

The current mainstay for GCAA management is anticoagulation and dual anti-platelet therapy (9). Anticoagulation is predominantly via vitamin K antagonists (VKAs) such as warfarin or low-molecular-weight heparin (LMWH) (9). However, these conventional approaches present considerable challenges in pediatric populations. Warfarin requires frequent international normalized ratio (INR) monitoring, and demonstrates substantial pharmacokinetic variability influenced by genetic polymorphisms, concurrent medications, and dietary vitamin K intake (10, 11). These factors often lead to suboptimal control. Similarly, LMWH necessitates subcutaneous administration, regular anti-factor Xa monitoring, and is not an appealing option to children and their parents, especially when long term therapy is required (11).

On the other hand, DOACs, including factor Xa inhibitors (rivaroxaban, apixaban, edoxaban) and direct thrombin inhibitors (dabigatran) offer predictable pharmacokinetics, minimal drug-food interactions, and obviate any laboratory monitoring (12, 13). Recent pediatric trials, extrapolated from adult experience, have demonstrated favorable efficacy and safety profiles for DOACs in venous thromboembolism (VTE) prevention and treatment (14–17). The latest American Heart Association (AHA) guidelines recommend anti-platelet and anticoagulant therapy for GCAAs (9, 18). Apart from conventional warfarin and LMWH, DOACs are now an option in KD management of giant aneurysms, despite lacking data comparing DOACs to traditional therapies. We sought to perform a comprehensive search to answer this.

This systematic review and meta-analysis aimed to comprehensively evaluate the comparative efficacy and safety profile of DOACs vs. conventional anticoagulation in children with KD-associated GCAAs. Many centers already use DOACs instead of conventional anticoagulants for GCAAs despite the lack of specific data. By synthesizing available evidence, we sought to inform clinical decision-making and expand therapeutic options in establishing treatment guidelines for this vulnerable population.

## 2 Methods

### 2.1 Protocol registration and reporting standards

This meta-analysis was conducted using data from published studies where ethical approval and informed consent were obtained by the original investigators. Therefore, no separate ethical approval was required. This systematic review and meta-analysis were conducted according to the Preferred Reporting Items for Systematic Reviews and Meta-Analyses (PRISMA) guidelines and registered prospectively in the International Prospective Register of Systematic Reviews (PROSPERO), PROSPERO 2025 CRD420251004094, available from https://www.crd.york.ac.uk/PROSPERO/view/CRD420251004094.

### 2.2 Search strategy

A comprehensive literature search was conducted independently by 2 authors (S.K.A.O. and D.R.) and executed across multiple databases from inception until October 2025. Databases searched included PubMed (MEDLINE), Embase, Cochrane Central Register of Controlled Trials and ClinicalTrials.gov. The detailed search strategy is available in Supplementary Table 1. The search was limited to studies that were published in English. The reference lists of included articles were also screened for additional articles.

### 2.3 Selection criteria

Studies were included if they met the following criteria: (1) enrolled patients younger than 19 years with documented KD-associated GCAAs, defined as coronary artery diameter ≥8 mm absolute dimension or Z-score ≥10 based on body surface area-adjusted normative data; (2) administered DOACs (rivaroxaban, apixaban, edoxaban, or dabigatran); (3) reported at least one efficacy outcome (coronary thrombosis, myocardial infarction, systemic thromboembolism) or safety outcome (major bleeding, clinically relevant non-major bleeding, CRNM).

Studies were excluded based on the following criteria: (1) studies including adult patients without separate pediatric data; (2) duplicate publications or overlapping patient cohorts; and (3) abstracts without full-text availability or insufficient outcome data.

### 2.4 Data collection and quality assessment

Two authors (S.K.A.O and D.R.) independently screened titles and abstracts based on the search results of the chosen databases, with potentially eligible studies undergoing full-text retrieval and review. Discrepancies were resolved through consensus or consultation with the third and fourth reviewers (Q.S.C. and K.P.L.). Data extraction was performed by two authors (S.K.A.O and D.R.) and utilized standardized forms capturing: study characteristics (design, setting, duration, country), sample size, patient demographics (age, time from KD diagnosis), intervention details (anticoagulant type, dosing, monitoring), as well as efficacy and safety outcomes. Discrepancies were mutually resolved. Attempts made to contact the primary authors of the included studies in order to obtain KD specific subgroup data were unsuccessful. Study quality was assessed using the Cochrane Risk of Bias tool (19).

### 2.5 Outcome definitions

The primary efficacy outcome was thromboembolic events (coronary thrombosis, myocardial infarction, systemic thromboembolism). Primary safety outcomes were major bleeding, defined according to International Society on Thrombosis and Haemostasis (ISTH) criteria: fatal bleeding, symptomatic bleeding in critical areas, or bleeding causing hemoglobin drop ≥2 g/dL or requiring ≥2 units blood transfusion, as well as CRNM (overt bleeding not meeting major criteria but requiring medical intervention).

Secondary outcomes were all bleeding events as well as serious adverse events (SAE) which were defined individually by each respective study.

Patient important bleeding no intervention events, such as epistaxis or subcutaneous haematoma, were classified as minor bleeding in this review.

### 2.6 Statistical analysis

Subgroup meta-analyses with a random effect model were performed to compare the primary outcomes between DOACs and control treatment within the main study period of the RCTs. The relative risk between DOACs and control treatment with 95% confidence interval (CI) for each event. Non-inferiority was claimed if the upper bound of the 95% CI for both efficacy and safety events were less than 1.2. In addition, all included studies (comprising non-RCTs as well) were used to estimate a pooled event rate of each outcome with 95% CI, and Freeman-Tukey transformation was applied.

All analyses were performed using Stata version 18 (StataCorp LLC) with two-tailed *p* values <0.05 considered statistically significant.

## 3 Results

### 3.1 Study identification and selection

**Figure 1:**
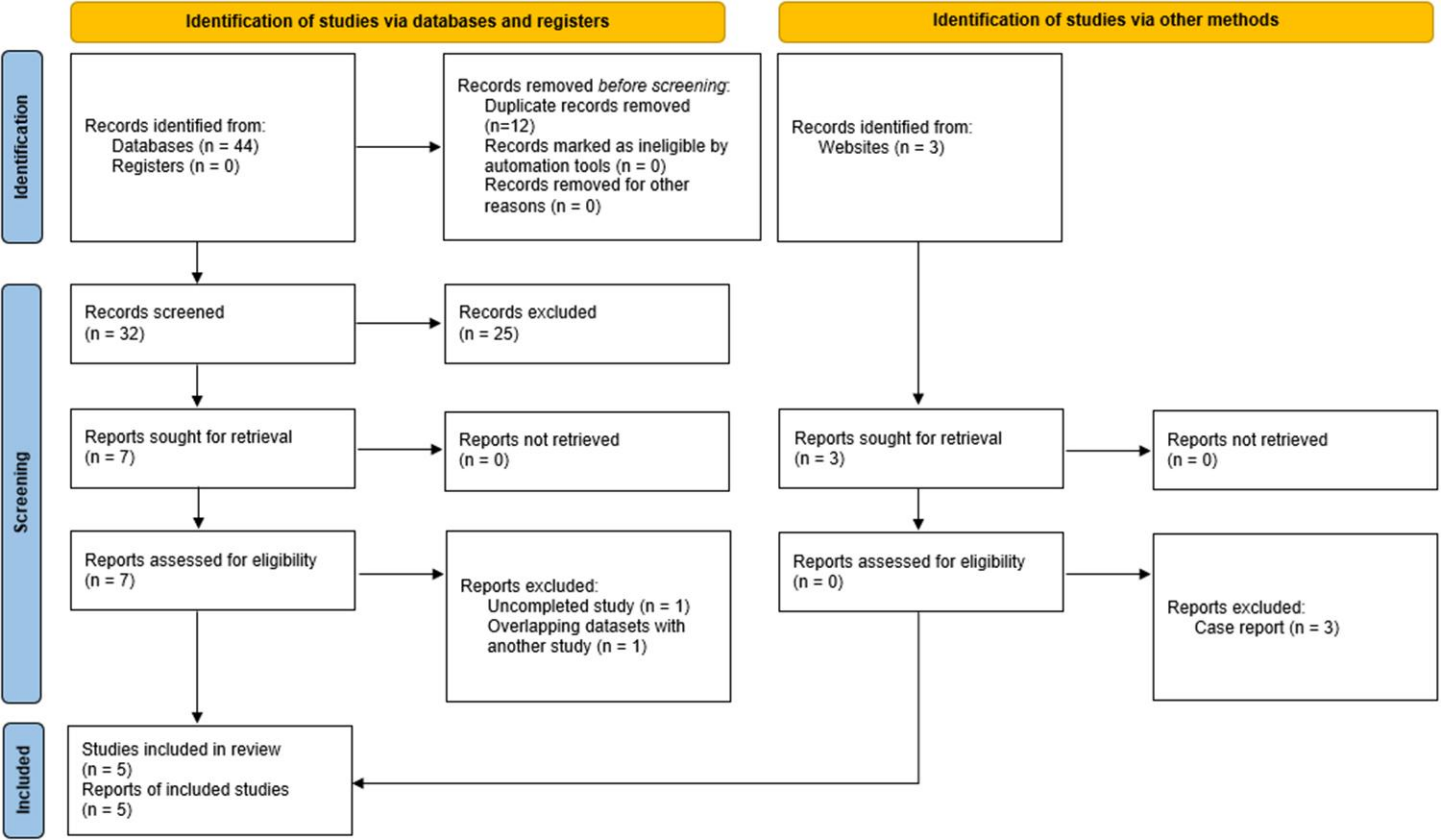
Study flow diagram of the study selection process.

The results of the search are reported in Figure 1. A total of 47 studies were identified, including 3 additional case reports (20–22) found via reference searching. Of the initial 47, 12 studies were excluded as they were duplicates. All remaining 35 study abstracts were reviewed with 25 studies excluded as they were irrelevant to the focus of our study. Of the remaining 10, the 3 case reports were not used for analysis (although they were taken into consideration by the authors). Two further studies were removed from analysis due to one being an ongoing study and the latter using an overlapping dataset. Eventually 5 studies (23–27) were included in the review.

Of the five included studies, two were randomized controlled trials (RCTs) (24, 25), one was a retrospective cohort study, one was a case series, and one was the most recently published prospective exploratory interventional study (23, 26, 27). Of note, although the patient population of the two RCTs encompassed patients with KD, the majority of the population required anticoagulation for other indications. The two RCTs were used for the meta-analysis while the rest of the studies were used to estimate a pooled event rate.

A total of 594 patients were included in our analysis: 474 (79.8%) patients were on treatment with DOACs while 120 (20.2%) were on standard of care (SOC). The study characteristics of the included studies are presented in Table 1.

**Table 1:** Study characteristics.

S/N	Author Name	Year of publication	Type of study	DOAC studied	Concomitant antiplatelet use	Age range	Mean age (y)	Total participants	Male	Number of participants on DOACs	Number of participants on SOC	Number of KD participants (DOAC/SOC)	Mean duration of exposure to DOACs (days)	Mean exposure to SOC (days)
1	Payne et al. (24)	2023	Clinical trial	Apixaban	43.6% on Aspirin	28d to 18y	7.8	188	102 (53.1%)	126	62	27 (16/11)	330.6	344.4
2	Portman et al. (25)	2022	Clinical trial	Edoxaban	Some on aspirin	Birth to 18y	8.1	167	109 (65.3%)	109	58	37 (24/13)	90	90
3	VanderPluym et al. (26)	2023	Retrospective cohort	Apixaban	60% on aspirin	110d to 19y	6.8	219	125 (57%)	219	N/A	13	162^a^	N/A
4	Dummer et al. (23)^b^	2023	Case series	Apixaban	Unknown	4y to 16y^a^	10.3	6	6 (100%)	6	N/A	6	2227	N/A
5	Dai et al. (27)	2025	Prospective interventional	Rivaroxaban	Aspirin	1 m to 18y	5.8^a^	14	10 (71%)	14	N/A	14	269.5^a^	N/A

DOAC, direct oral anticoagulant; KD, Kawasaki disease; SOC, standard of care.

^a^ Reported median values (mean values were not reported in the respective studies).

^b^ There were a total of 24 patients age 4–67 years old in the case series, but only patients <18 years old (total of 6 patients) from this paper were included and analysed.

### 3.2 Risk of bias

Methodological quality was assessed using the Cochrane Risk of Bias tool for RCTs. Key domains evaluated included selection bias, comparability of cohorts, outcome assessment, and completeness of follow-up. Studies were categorized as low, moderate, or high risk of bias.

Out of the five studies analysed, no study was identified to be low risk. Payne et al. and Portman et al. were both considered to be moderate risk in view of their open-label design but scored well in the other measures (24, 25). The inclusion of Dummer et al. (case series), VanderPluym et al. (retrospective cohort) and Dai et al. (prospective interventional) contribute to a higher risk of bias (23, 26, 27). Being non-randomised designs, they were particularly vulnerable to selection bias and unmeasured confounding factors.

An overall holistic analysis of the five studies revealed a moderate level of bias across all domains cumulatively (Supplementary Figure 1).

### 3.3 Outcomes

The results of the primary and secondary outcomes are presented in Table 2. For purpose of analysis, the results were split into efficacy (thromboembolic events) and safety (bleeding and SAEs) outcomes.

**Table 2:** Primary and secondary outcomes.

S/N	Name	Efficacy outcomes		Safety outcomes					
		Thromboembolic events with		Major and/or CRNM bleeding with		All bleeding events with		Serious adverse events with	
		DOACs	SOC	DOACs	SOC	DOACs	SOC	DOACs	SOC
1	Payne et al. (24)	0	0	1	3	47	23	26	13
2	Portman et al. (25)	0	1	1	1	4	2	5	3
3	VanderPluym et al. (26)	0	N/A	4	N/A	22	N/A	7	N/A
4	Drummer et al. (23)	0	N/A	0	N/A	0	N/A	Unknown	N/A
5	Dai et al. (27)^a^	0	N/A	0	N/A	2	N/A	Unknown	N/A

DOACs, direct oral anticoagulant; SOC, standard of care; CRNM, clinically relevant non-major; N/A, not available.

^a^ Outcomes were only obtained from the second phase of the trial as there were 4 CRNM bleeding events in 3 patients during the first phase necessitating dose adjustments.

#### 3.3.1 Efficacy outcomes

**Figure 2:**
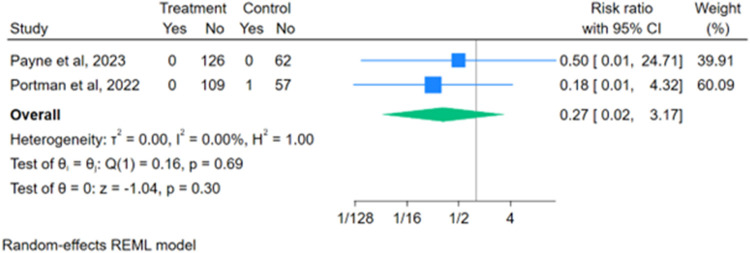
Forest plot for thromboembolic events.

There were no reported thromboembolic or coronary artery thrombosis events for patients on DOACs within the study period of the 5 studies and only 1 recorded event for a patient on SOC within the two RCTs. The overall risk ratio of thromboembolic events for patients on DOACs vs. SOC was not statistically significant [RR 0.27 (95% CI: 0.02–3.17); *p* = 0.29] (Figure 2).

#### 3.3.2 Safety outcomes

**Figure 3:**
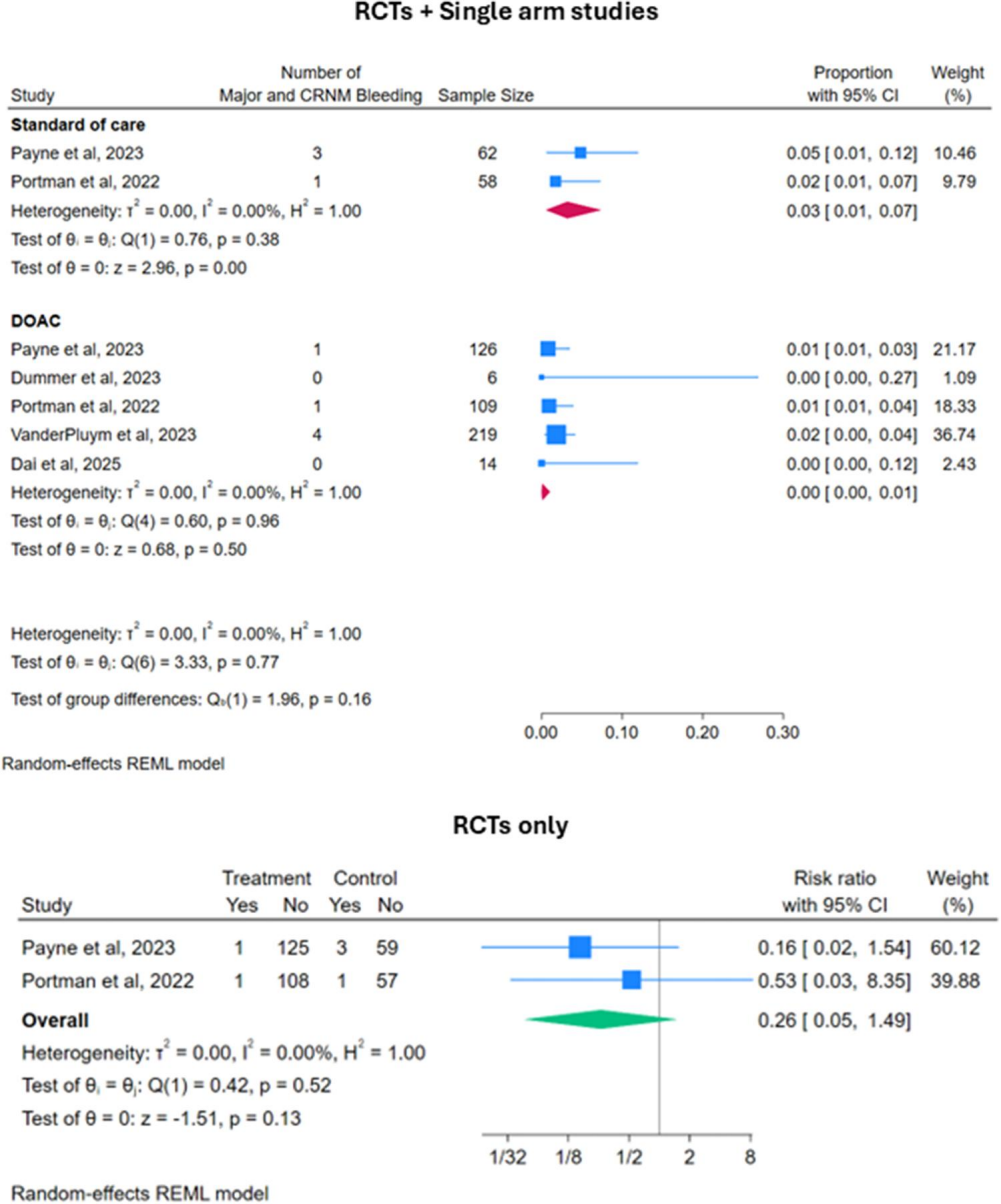
Forest plots for safety outcomes: major or clinically relevant non-major (CRNM) bleeding events.

There was a total of 6 major or CRNM bleeding events for patients on DOACs recorded across the 5 studies while there were 4 events for patients on SOC recorded in the two RCTs. The estimated pooled major or CRNM bleeding event rate was similar for patients on DOACs [Proportion 0.001 (95% CI: 0.00–0.01)] vs. patients on SOC [Proportion 0.03 (95% CI: 0.01–0.07)]. Taking into consideration both the RCTs, although the point estimate of the relative risk was less than 1 in the DOACs population (Figure 3), the estimated risk ratio between DOACs vs. SOC was not statistically significant [RR 0.26 (95% CI: 0.05–1.49); *p* = 0.13].

**Figure 4:**
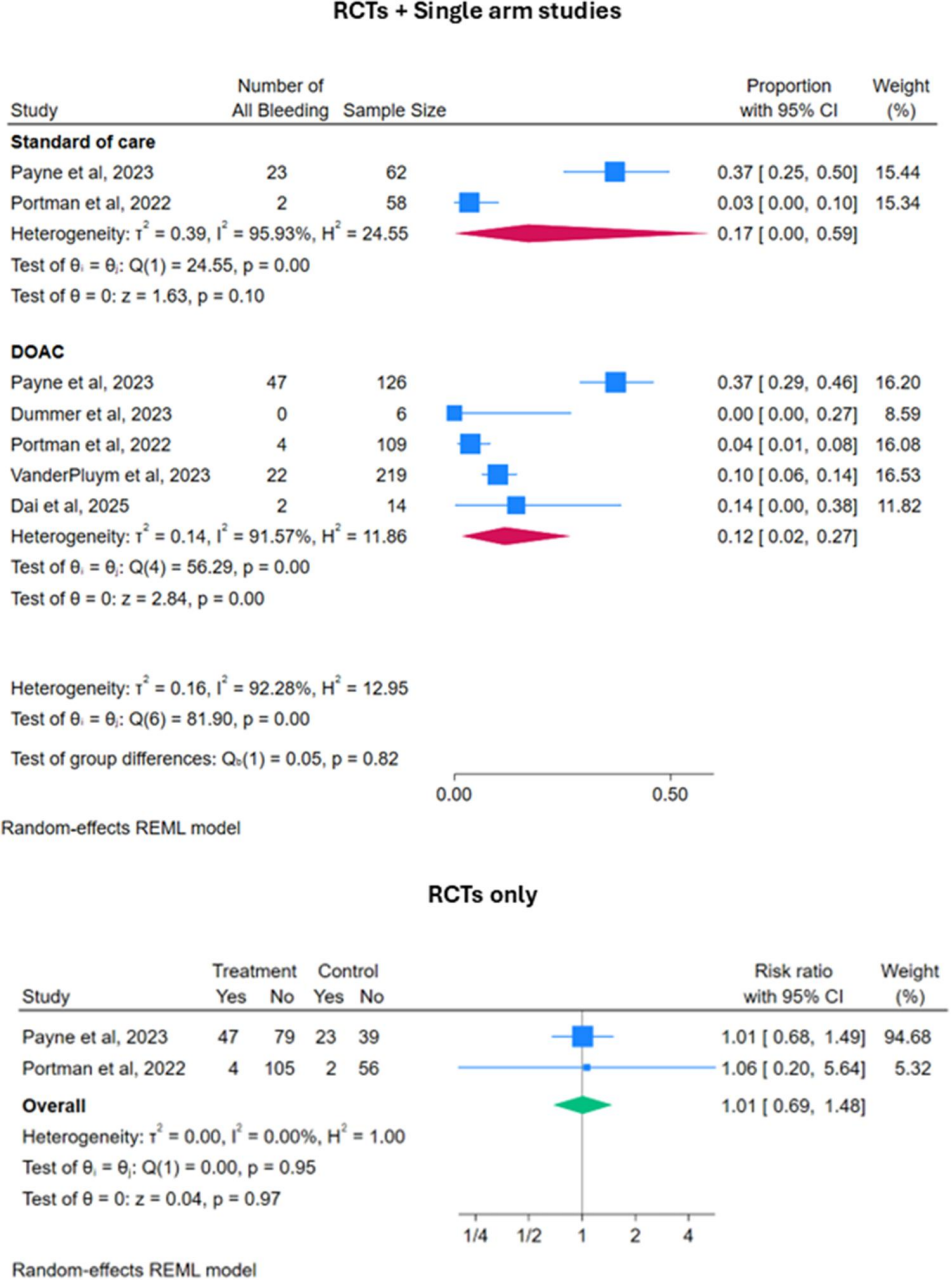
Forest plots for safety outcomes: all bleeding events.

Regarding secondary outcomes, the estimated pooled all bleeding event rate for patients on DOACs [Proportion 0.12 (95% CI: 0.02–0.27)] was similar to patients on SOC [Proportion 0.17 (95% CI: 0.00–0.59)]. When analysing the two RCTs alone, there was no significant difference in the risk of all bleeding between the two groups [RR 1.01 (95% CI: 0.69–1.48); *p* = 0.97] (Figure 4).

**Figure 5:**
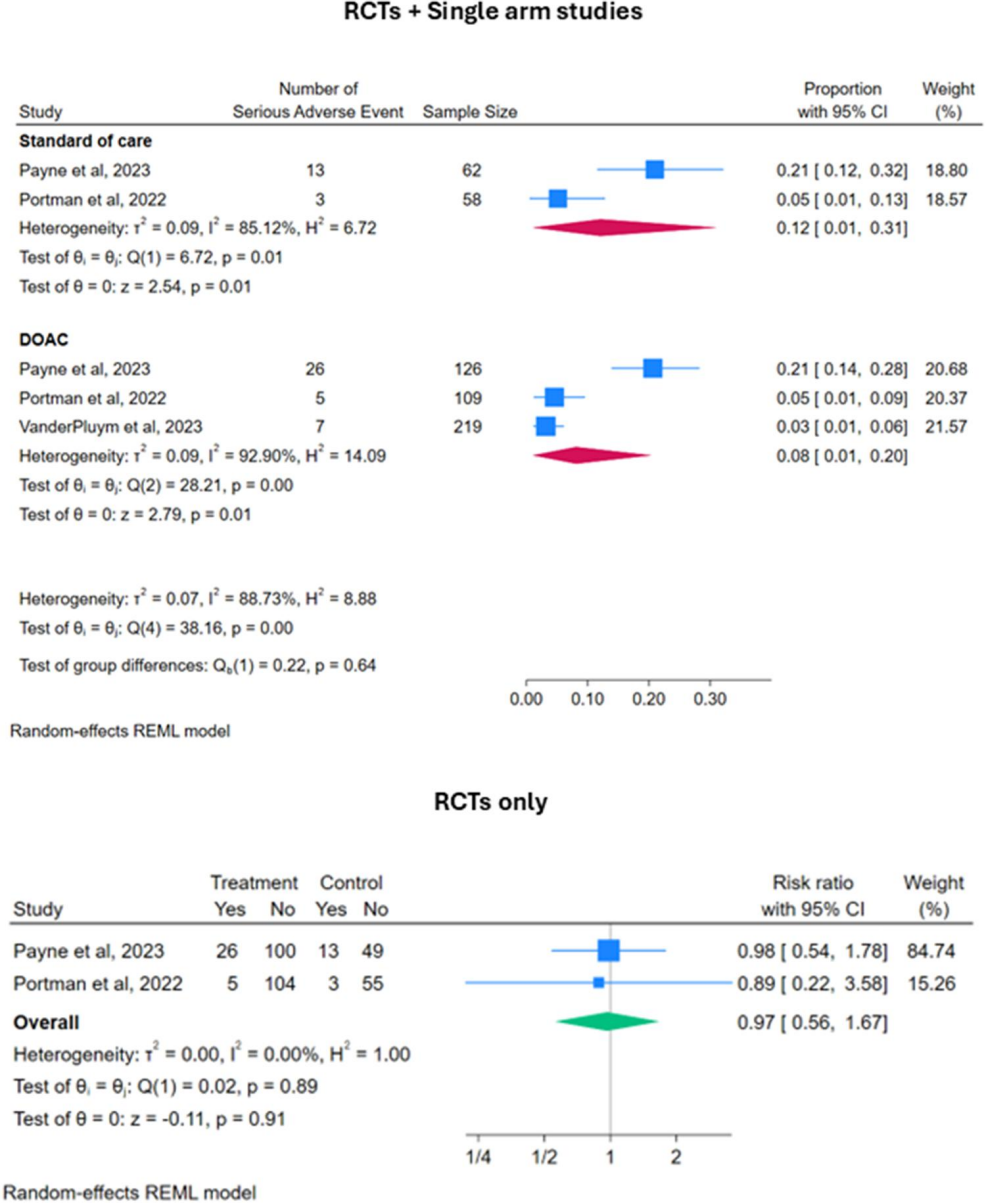
Forest plots for safety outcomes: serious adverse events.

Only 3 of the 5 studies reported SAE rates and were therefore included in the corresponding analysis. The table of SAEs can be found in Supplementary Table 2. The estimated pooled SAE risk for patients on DOACs [Proportion 0.08 (95% CI: 0.01–0.20)] was similar to patients on SOC [Proportion 0.12 (95% CI: 0.01–0.31)]. In addition, the estimated risk ratio was not significant [RR 0.97 (95% CI: 0.56–1.67); *p* = 0.90] when analysing the two RCTs (Figure 5).

## 4 Discussion

To date, this is the first meta-analysis that directly addresses and analyses the use of DOACs for GCAAs specific to KD.

The non-inferiority outcomes reported in this review put DOACs on par with conventional medications as a first-line anticoagulation strategy for the paediatric population, including children with Kawasaki disease-associated GCAAs, where drug monitoring and administration are important considerations. The work will be of interest to clinicians, researchers, and policymakers in shaping evidence-based guidelines in the management of GCAAs from KD.

The practical advantages of DOAC therapy are particularly relevant for long-term pediatric management. Avoidance of routine laboratory monitoring and subcutaneous injections may reduce treatment burden, improve adherence, and enhance quality of life (8, 11, 27). The predictable dose-response profile of DOACs may also offer stability during periods of growth requiring weight-based dose adjustments (8, 29).

Despite these encouraging findings, the absence of randomized comparative trials and the limited size of existing cohorts necessitate cautious interpretation. Larger prospective studies are required to more definitively establish the role of DOACs in KD-associated coronary arteriopathy.

While our findings support DOACs use in KD-associated GCAAs, several research priorities emerge. Long term outcome data beyond 5 years remain limited and potentially not all patients respond to DOACs, despite preliminary promising results from the RCTs. For example, as reported in one of the conference abstracts (20), there is a single case of an 18-year-old male with GCAA from childhood KD with extensive thrombus burden who switched from warfarin to a DOAC after his first myocardial infarction (MI) and developed multiple MIs over the course of 6 months (while on DOACs) before switching back to warfarin. Thereafter, he remained stable and event-free for 3 years (20). The authors acknowledge that although this was a stand-alone case in a complex KD patient predisposed to MI, vigilance is prudent, especially in those with pre-existing high thrombus burden.

In addition to the case report, it is important to address that the ENNOBLE-ATE trial (25) did report 4 TE events (one of which was deemed to be a haemorrhagic stroke instead of a thromboembolic stroke) during the extension period. These TE events were not included in the current meta analysis because the extension period involved all patients in both edoxaban and SOC arms switching to edoxaban for the subsequent 3–12 month period. However, despite not including these events into our study, these events support the fact that long term follow-up data is desperately needed as it may impact our understanding of the efficacy and safety outcomes of DOACs.

A critical knowledge gap also remains regarding concomitant bleeding risks for patients who are on concurrent antiplatelet therapy. The use of antiplatelet agents was not consistently reported across the five papers and hence not able to be analysed as part of this study. As many patients with GCAAs receive concurrent antiplatelet agents, the specific bleeding risk-benefit profile of DOAC-antiplatelet combinations also needs to be studied (1).

The potential adoption of DOACs for GCAA management carries important global health implications. In resource-limited settings where access to laboratory monitoring for warfarin therapy is challenging, DOACs could improve treatment accessibility and outcomes. The simplified dosing and monitoring requirements of DOACs may facilitate care delivery in regions with limited pediatric cardiology expertise, potentially reducing geographic disparities in KD outcomes (28).

Although DOACs are more expensive than warfarin in terms of direct drug cost, the reduction in monitoring requirements and the associated logistical burden for both patients and healthcare systems—coupled with quality-of-life advantages and the potential for improved adherence—likely more than offset the higher cost (29).

Future research should focus on optimizing pediatric-specific dosing strategies, evaluating long-term outcomes, and developing risk stratification tools to personalize anticoagulation management. As evidence continues to accumulate, DOACs are poised to become an integral component in the therapeutic armamentarium for managing GCAAs in KD, offering hope for improved quality of life and clinical outcomes for affected children and their caregivers worldwide.

### 4.1 Limitations

The incidence of GCAAs among children with KD is low. The limited number of included RCTs—neither of which was adequately powered for superiority or non-inferiority—necessitated the inclusion of observational studies to strengthen the analysis. Moreover, the low event rates for the primary outcomes, particularly thromboembolic and major bleeding events, likely contributed to reduced statistical power and, consequently, non-significant results.

Heterogeneity in the types of DOACs used and the definitions of outcomes across studies may limit the generalisability of the pooled estimates. Furthermore, the relatively short follow-up durations in most studies preclude evaluation of late thrombotic complications and long-term safety outcomes.

### 4.2 Conclusions

This systematic review and meta-analysis demonstrates that DOACs may have similar efficacy and safety profiles compared to SOC in patients with KD-associated GCAAs. In addition, the well-established favourable administration characteristics with minimal need for laboratory surveillance are significant advantages. Together, these findings support the potential for DOACs as a first-line anticoagulation strategy in children with high risk of thromboembolic events. Larger scale randomised trials targeted at the KD population with longer follow-up are useful to confirm this, and can potentially transform the therapeutic landscape for one of childhood's most serious cardiovascular conditions.

## Data Availability

The original contributions presented in the study are included in the article/Supplementary Material, further inquiries can be directed to the corresponding author.
