# Supplementary material for: Direct oral anticoagulants in children with giant coronary artery aneurysms from Kawasaki disease: a systematic review and meta-analysis
Source: Front Cardiovasc Med. 2026 Apr 10;13:1777856. doi: 10.3389/fcvm.2026.1777856 (PMC13106334; doi:10.3389/fcvm.2026.1777856)
Supplement: Supplementary file 1 [file Datasheet1.docx]

Supplementary Material

# Supplementary Figures and Tables

## Supplementary Figures

**
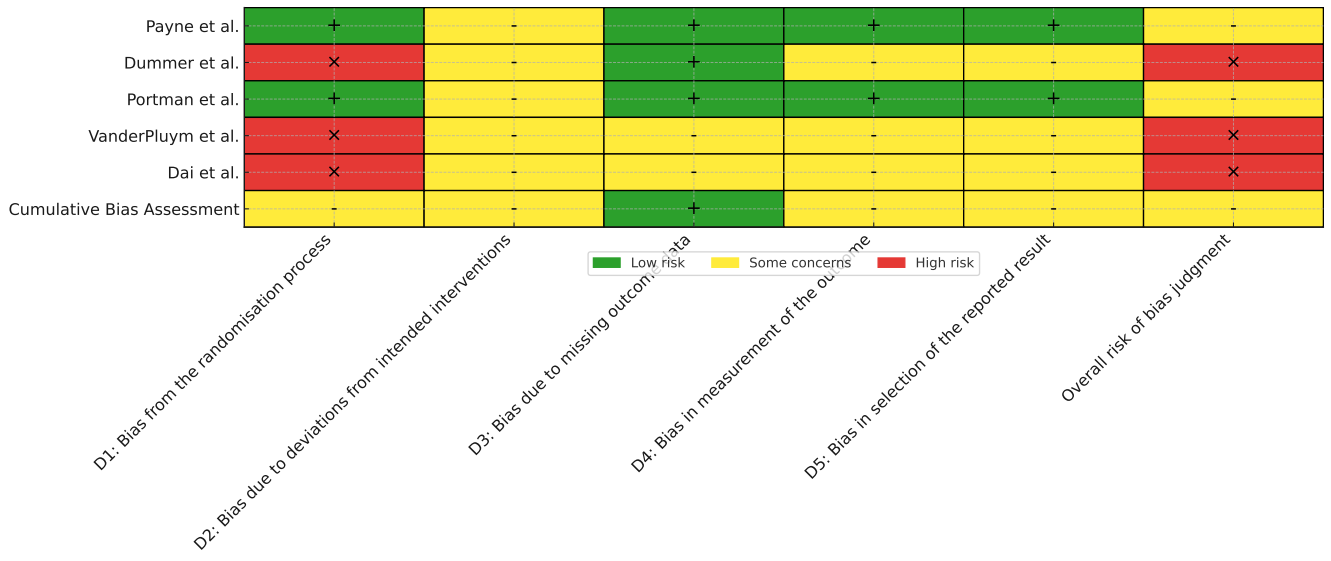
**

**Supplementary Figure 1.** Risk of bias assessment.

## Supplementary Tables

**Supplemental Table 1**. Search Strings

| **PUBMED** |
| --- |
| (("Kawasaki Disease"[Mesh] OR "Kawasaki disease"[tiab] OR "mucocutaneous lymph node syndrome"[tiab]))AND( ("Direct Oral Anticoagulants"[Mesh] OR "direct oral anticoagulant"[tiab] OR "direct oral anticoagulants"[tiab] OR "DOAC"[tiab] OR "DOACs"[tiab] OR "non-vitamin K antagonist oral anticoagulant"[tiab] OR "non-vitamin K antagonist oral anticoagulants"[tiab] OR "NOAC"[tiab] OR "NOACs"[tiab] OR "direct factor Xa inhibitor"[tiab] OR "direct thrombin inhibitor"[tiab] OR apixaban[tiab] OR rivaroxaban[tiab] OR edoxaban[tiab] OR dabigatran[tiab] OR betrixaban[tiab]))AND  ((infant[MeSH] OR child[MeSH] OR adolescent[MeSH] OR "pediatric"[tiab] OR "paediatric"[tiab] OR "child"[tiab] OR "children"[tiab] OR "infant"[tiab] OR "infants"[tiab] OR "adolescent"[tiab] OR "adolescents"[tiab] OR "teenager"[tiab] OR "teenagers"[tiab])) |
| **EMBASE** |
| ('kawasaki disease'/exp OR 'kawasaki disease':ti,ab,kw OR 'mucocutaneous lymph node syndrome':ti,ab,kw)AND( 'direct oral anticoagulant'/exp OR 'direct oral anticoagulant':ti,ab,kw OR 'direct oral anticoagulants':ti,ab,kw OR DOAC:ti,ab,kw OR DOACs:ti,ab,kw OR 'non-vitamin K antagonist oral anticoagulant':ti,ab,kw OR 'non-vitamin K antagonist oral anticoagulants':ti,ab,kw OR NOAC:ti,ab,kw OR NOACs:ti,ab,kw OR 'direct factor Xa inhibitor':ti,ab,kw OR 'direct thrombin inhibitor':ti,ab,kw OR apixaban:ti,ab,kw OR rivaroxaban:ti,ab, kw OR edoxaban:ti,ab,kw OR dabigatran:ti,ab,kw OR betrixaban:ti,ab,kw)AND( 'child'/exp OR 'adolescent'/exp OR 'infant'/exp OR pediatric:ti,ab,kw OR paediatric:ti,ab,kw OR child:ti,ab,kw OR children:ti,ab,kw OR infant:ti,ab,kw OR infants:ti,ab,kw OR adolescent:ti,ab,kw OR adolescents:ti,ab,kw OR teenager:ti,ab,kw OR teenagers:ti,ab,kw) |
| **COCHRANE** |
| ("Kawasaki Disease" OR "mucocutaneous lymph node syndrome") AND ("direct oral anticoagulant" OR "direct oral anticoagulants" OR DOAC OR DOACs OR "non-vitamin K antagonist oral anticoagulant" OR "non-vitamin K antagonist oral anticoagulants" OR NOAC OR NOACs OR "direct factor Xa inhibitor" OR "direct thrombin inhibitor" OR apixaban OR rivaroxaban OR edoxaban OR dabigatran OR betrixaban)  AND (pediatric OR paediatric OR child OR children OR infant OR infants OR adolescent OR adolescents OR teenager OR teenagers) |

**Supplemental Table 2**. Serious Adverse Events (SAEs) per study (excluding thrombosis and bleeding events)

| **S/N** | **Name** | **Serious Adverse Event (SAEs) Definition** | **SAEs on DOAC** | **Treatment-related SAEs** | **Discontinuation due to SAE** | **Types of SAEs** | | | | | | |
| --- | --- | --- | --- | --- | --- | --- | --- | --- | --- | --- | --- | --- |
|  |  |  |  |  |  | **Arrhythmia** | **Contusion** | **COVID-19** | **Influenza** | **Pleural effusion** | **Protein-losing gastroenteropathy** | **Cardiac failure** |
| 1 | Payne et al. | SAEs were included if experienced within 30 days following the last dose. | 26 | 6 | 4 | 1 | 1 | 1 | 1 | 1 | 1 |  |
| 2 | Portman et al. | SAEs in this study were defined as “subjects with at least 1 serious TEAE”. | 5 | 0 | Unknown |  |  |  |  | 2 |  | 2 |
| 3 | VanderPluym et al. | SAEs in this study included 3 deaths of cardiac (non-hematologic/non-thrombotic/non-haemorrhagic) causes. | 3 | 0 | Unknown | 1 |  |  |  |  |  | 2 |
| 4 | Drummer et al. | No non-bleeding/thrombosis SAEs defined | 3 | Unknown | Unknown |  |  |  |  |  |  |  |
